# Hidden endemism, deep polyphyly, and repeated dispersal across the Isthmus of Tehuantepec: Diversification of the White‐collared Seedeater complex (Thraupidae: *Sporophila torqueola*)

**DOI:** 10.1002/ece3.3799

**Published:** 2018-01-12

**Authors:** Nicholas A. Mason, Arturo Olvera‐Vital, Irby J. Lovette, Adolfo G. Navarro‐Sigüenza

**Affiliations:** ^1^ Department of Ecology and Evolutionary Biology Cornell University Ithaca NY USA; ^2^ Fuller Evolutionary Biology Program Cornell Laboratory of Ornithology Ithaca NY USA; ^3^ Museo de Zoología Facultad de Ciencias UNAM México Mexico

**Keywords:** bird, endemism, Mesoamerica, phylogeography, taxonomy

## Abstract

Phenotypic and genetic variation are present in all species, but lineages differ in how variation is partitioned among populations. Examining phenotypic clustering and genetic structure within a phylogeographic framework can clarify which biological processes have contributed to extant biodiversity in a given lineage. Here, we investigate genetic and phenotypic variation among populations and subspecies within a Neotropical songbird complex, the White‐collared Seedeater (*Sporophila torqueola*) of Central America and Mexico. We combine measurements of morphology and plumage patterning with thousands of nuclear loci derived from ultraconserved elements (UCEs) and mitochondrial DNA to evaluate population differentiation. We find deep levels of molecular divergence between two *S. torqueola* lineages that are phenotypically diagnosable: One corresponds to *S. t. torqueola* along the Pacific coast of Mexico, and the other includes *S. t. morelleti* and *S. t. sharpei* from the Gulf Coast of Mexico and Central America. Surprisingly, these two lineages are strongly differentiated in both nuclear and mitochondrial markers, and each is more closely related to other *Sporophila* species than to one another. We infer low levels of gene flow between these two groups based on demographic models, suggesting multiple independent evolutionary lineages within *S. torqueola* have been obscured by coarse‐scale similarity in plumage patterning. These findings improve our understanding of the biogeographic history of this lineage, which includes multiple dispersal events out of South America and across the Isthmus of Tehuantepec into Mesoamerica. Finally, the phenotypic and genetic distinctiveness of the range‐restricted *S. t. torqueola* highlights the Pacific Coast of Mexico as an important region of endemism and conservation priority.

## 1 INTRODUCTION

Variation is ubiquitous in nature. Yet lineages differ in how phenotypic and genetic diversity are partitioned among individuals, populations, and species. This complexity motivates long‐standing efforts to identify the evolutionary and ecological forces that generate and maintain biodiversity across temporal and spatial scales. Phylogeography quantifies genetic and phenotypic partitioning within and among species to provide insight into the patterns and processes that underlie population differentiation, speciation, and diversification (Avise, Arnold, Ball, & Bermingham, 1987; Brito & Edwards, 2008; Edwards, Potter, Schmitt, Bragg, & Moritz, 2016). Examining concordance (or lack thereof) between genetic and phenotypic partitions in phylogeographic data sets can reveal the contributions of historical demographic events and selective pressures toward extant patterns of biodiversity (García‐Moreno, Navarro‐Sigüenza, Peterson, & Sánchez‐González, 2004; Zamudio, Bell, & Mason, 2016), while large genomic data sets continue to provide new insights into the origins of biodiversity (Ekblom & Galindo, 2010; McCormack, Hird, Zellmer, Carstens, & Brumfield, 2013; Toews, Campagna, et al., 2016).

The Neotropics are remarkably diverse (Orme et al., 2005; Smith, McCormack, et al., 2014), and numerous studies of biodiversity in Central and South America have generated insights into the roles of mountain orogeny (Cadena, Klicka, & Ricklefs, 2007; Chaves, Weir, & Smith, 2011; Sedano & Burns, 2010; Winger & Bates, 2015), riverine barriers (Cracraft, 1985; Naka, Bechtoldt, & Henriques, 2012; Weir, Faccio, Pulido‐Santacruz, Barrera‐Guzmán, & Aleixo, 2015), glacial cycles (Turchetto‐Zolet, Pinheiro, Salgueiro, & Palma‐Silva, 2012; Vuilleumier, 1971; Weir, 2006), and transcontinental dispersal (Smith, Amei, & Klicka, 2012; Smith & Klicka, 2010; Weir, Bermingham, & Schluter, 2009) in shaping regional and continental biotas. Within the Neotropics, phylogeographic and biogeographic studies have primarily focused on the hyperdiverse biotic assemblages of the Andes Mountains (Benham, Cuervo, Mcguire, & Witt, 2015; Chaves & Smith, 2011; Gutiérrez‐Pinto et al., 2012; Isler, Cuervo, Gustavo, & Brumfield, 2012; Valderrama, Pérez‐Emán, Brumfield, Cuervo, & Cadena, 2014; Winger et al., 2015) and lowland Amazonia (Aleixo, 2004, 2006; Cheviron, Hackett, & Capparella, 2005; Fernandes, Wink, & Aleixo, 2012; Harvey & Brumfield, 2015; Matos et al., 2016), while substantially fewer studies have examined continental phylogeographic patterns in Central America and Mexico (Beheregaray, 2008). While various recent studies have improved our understanding of phylogeographic patterns for certain birds in Mesoamerica (Battey & Klicka, 2017; DaCosta & Klicka, 2008; González, Ornelas, & Gutiérrez‐Rodríguez, 2011; Lavinia et al., 2015; Miller, Bermingham, & Ricklefs, 2007; Núñez‐Zapata, Peterson, & Navarro‐Sigüenza, 2016; Ortiz‐Ramírez, Andersen, Zaldívar‐Riverón, Ornelas, & Navarro‐Sigüenza, 2016), the lack of data for many taxa has precluded a comprehensive understanding of the biogeographic history of this region (Peterson & Navarro‐Sigüenza, 2016).

**Figure 1:**
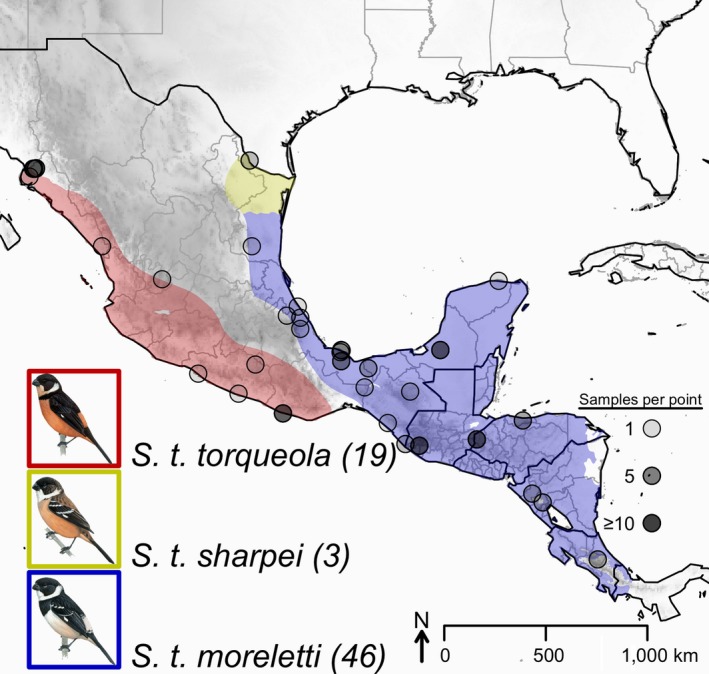
Map showing sampling localities of samples used in this study. Sampling points have been jittered to help visualize the density of samples at localities with more than one sample. Sample numbers are shown next to each species’ portrait. See Table S1 for more detailed information on sampling localities used in genetic analyses

In this study, we examine phenotypic and genetic variation among populations and subspecies within the White‐collared Seedeater (*Sporophila torqueola*) complex. The *S. torqueola* complex—and other members of the genus *Sporophila*—includes small‐bodied, granivorous songbirds with striking plumage dimorphism that occupy open areas, including savannas, marsh edges, pastures, and roadsides in the Neotropics (Hilty, 2011). Molecular phylogenetic studies have established that *S. torqueola* and its congeners are tanagers (Thraupidae), which constitute a widespread and phenotypically diverse family of songbirds (Burns, Unitt, & Mason, 2016; Burns et al., 2014; Lijtmaer, Sharpe, Tubaro, & Lougheed, 2004). However, the phylogenetic placement of *S. torqueola* within the subfamily Sporophilinae remains uncertain (Mason & Burns, 2013). Most taxonomic authorities have long recognized between three and five geographically restricted subspecies within *S. torqueola*, which differ primarily in male plumage patterning and coloration (Clements et al., 2016; Gill & Donsker, 2017). Here, we measure bill morphology, body size and shape, and plumage characters of vouchered specimens to evaluate geographic variation among three subspecies groups: (1) *S. t. torqueola* and *S. t. atriceps* (hereafter *S. t. torqueola*), which occur in the Pacific lowlands of Mexico, from Sinaloa southeast to Guanajuato and Oaxaca; (2) *S. t. morelleti* and *S. t. mutanda* (hereafter *S. t. morelleti*), which occur in the Caribbean coastal lowlands of Mexico from Veracruz south through Central America to western Panama; and (3) *S. t. sharpei*, which occurs along the coastal Gulf of Mexico from southern Texas to Veracruz (Figure 1). We also use a large panel of molecular markers to examine how genetic variation is partitioned among individuals and populations within this complex. Finally, we consider the biogeographic history of the *S. torqueola* complex in the broader phylogenetic context of related taxa in the Neotropical subfamily Sporophilinae.

## 2 METHODS

### 2.1 Morphological measurements

We measured standard phenotypic characters to examine geographic and taxonomic variation in morphology and plumage. We recorded a set of standard measurements (culmen length, bill length from the gonys, depth of the bill at the nostril, width of the bill at the nostril, wing chord length, tarsus length, hallux length, and length of the central rectrix). We also assessed multiple presence–absence plumage characters, including a partial eye ring, primary wing bars, and white edging on the secondaries and tertials of the wing feathers, and a white spot at the base of the primaries. Measurements and plumage scores of 663 specimens of *S. torqueola*, including at least 25 individuals of both sexes from all three groups (Appendix S1), were all conducted by the same observer (Olvera‐Vital). We performed a principal component analysis (PCA) and examined the dispersion of measured specimens corresponding to the three groups for both males and females. We also conducted a multinomial logistic regression to evaluate the diagnosability of each group using both the continuous morphological measurements and presence–absence plumage characters.

### 2.2 Sampling, DNA extraction, target capture, and sequencing

We sampled 68 vouchered specimens for molecular analyses, including representatives of all three groups within *S. torqueola* (Figure 1; Table S1). Our sampling also included four *S. minuta* individuals as an outgroup (Mason & Burns, 2013). We extracted genomic DNA from tissues or museum specimen toe pads using a Qiagen DNeasy Blood and Tissue kit. We quantified each extraction using a QuBit fluorometer (Life Technologies, Inc.), diluted or concentrated each sample to approximately 20 ng/μl, and visualized genomic DNA on an agarose gel to assess potential degradation.

We performed sequence capture of ultraconserved elements (UCEs) to acquire a reduced‐representation genomic data set (Mamanova et al., 2010). This method captures DNA fragments from thousands of orthologous loci using synthetic probes that were generated from alignments of amniote genomes, including chicken and *Anolis* (Faircloth et al., 2012). Once orthologous loci have been successfully captured and recovered, single nucleotide polymorphisms and indels that are present in regions adjacent to the UCEs are called for use in downstream analyses. While UCEs were first used to resolve deeper evolutionary relationships (Crawford et al., 2012; McCormack et al., 2012; McCormack, Harvey, et al., 2013), various studies have demonstrated their potential to infer evolutionary processes within more recent evolutionary time scales (Giarla & Esselstyn, 2015; Harvey, Smith, Glenn, Faircloth, & Brumfield, 2016; Smith, Harvey, Faircloth, Glenn, & Brumfield, 2014).

We processed 24 of our samples using published, open‐source protocols for library preparation and UCE enrichment (Faircloth et al., 2012; http://ultraconserved.org). In brief, we sheared 100 μl of each DNA extraction after normalizing concentrations to a distribution of 200–400 bp using a Bioruptor sonicator (Diagenode). We did not shear degraded samples, including extractions from toepads of museum specimens. We constructed genomic libraries for each sample using KAPA LTP library preparation kits with custom PCR barcodes (Faircloth & Glenn, 2012). We pooled indexed libraries into groups of eight and enriched each pool using a set of 5,472 probes that targeted 5,060 UCE loci (Tetrapods‐UCE‐K5v1 probe set; http://ultraconserved.org). We performed an 18‐cycle PCR‐recovery and combined libraries in equimolar ratios to achieve a final concentration of 10 μM. We send the pooled libraries to the UCLA Genoseq Facility for 250 bp paired‐end sequencing on one lane of an Illumina MiSeq. For the remaining 44 samples, we sent DNA extractions to RAPiD Genomics (http://www.rapid-genomics.com) for enrichment of the same probe set to be run on 25% of a shared, single lane of Illumina HiSeq2000.

### 2.3 Bioinformatic processing of UCEs and mtDNA

We followed a modified version of the PHYLUCE bioinformatic pipeline (Faircloth, 2016) that was optimized for shallow‐scale population genetic and phylogenetic downstream analyses (see https://github.com/mgharvey/seqcap_pop; Harvey et al., 2016). In short, we first cleaned raw reads with Trimmomatic (Bolger, Lohse, & Usadel, 2014). Next, we assembled reads into contigs using Velvet (Zerbino, 2002) in combination with VelvetOptimiser (Zerbino & Birney, 2008), which tested the assembly performance of hash lengths that varied from 67 to 75. Following contig assembly, we aligned reads to probes and their corresponding contigs using BWA (Li & Durbin, 2009), created indexed bam files with SAMtools (Li et al., 2009), and called phased SNPs and indels via GATK (DePristo et al., 2011; McKenna et al., 2010). We retained only high quality SNPs (Phred Score ≥ Q30) and prepared input files for downstream population genetic analyses.

Mitochondrial DNA is often present among postcapture libraries due to off‐target capture (Mamanova et al., 2010). We assembled mtDNA sequences using the program MITObim (Hahn, Bachmann, & Chevreux, 2013), which uses an iterative baiting method to generate mtDNA contigs. We used a published sequence of cytochrome *b* (cyt b) as our initial reference (GenBank accession JN810151). To examine the evolutionary relationships of the samples included this study with respect to other species in the *Sporophila* genus, we also downloaded existing cyt b sequences for other species (Burns et al., 2014; Mason & Burns, 2013). We aligned our cyt b assemblies with MAFFT (Katoh & Standley, 2013) for downstream analyses.

### 2.4 Phylogenetic and population genetic analyses

We first conducted phylogenetic analyses on all individuals using the UCE data set. For the phylogenetic analyses, we extracted the 1,000 UCE loci with the highest number of informative sites using AMAS (Borowiec, 2016) and custom R scripts. We used RAxML (Stamatakis, 2006) with the “‐f a” run option that finds the best tree and performs rapid bootstrapping in the same run with a separate partition for each UCE locus. We performed the same RAxML analyses on the cyt b alignments of mtDNA. We ran these analyses on an alignment matrix that included outgroup individuals and one that omitted outgroup individuals.

We then performed a Discriminant Analysis of Principal Components (DAPC; Jombart, Devillard, & Balloux, 2010) on the data set with outgroups as well as with outgroups removed using the adegenet package in R (Jombart & Ahmed, 2011). For the data set with outgroups, we removed individuals with more than 85% missing data and loci that had more than 25% missing data to generate a data matrix of 54 individuals and 1,358 loci. For the data set without outgroups, we removed individuals with more than 80% missing data and loci with more than 75% missing data to generate a matrix of 50 individuals and 4,067 loci. For both data sets, we retained principal component axes that together accounted for at least 70% of the total variation and constructed a scatterplot of the first two principal component axes to examine population structure.

We ran STRUCTURE (Pritchard, Stephens, & Donnelly, 2000) on the full data matrix to evaluate different population assignment models. We ran STRUCTURE with different *K* values (i.e., number of populations) from 1 to 6 with 10 replicate runs for each *K* value. Each run included 500,000 generations preceded by 10,000 burn‐in steps in the MCMC chain. We identified the optimal *K* value by examining changes in log‐likelihood scores and variance in log‐likelihoods among runs (Evanno, Regnaut, & Goudet, 2005). After identifying the optimal *K* value for the full data matrix, we ran STRUCTURE with the same settings on the data matrix with outgroups removed and separately on two prominent clusters identified in our data to examine possible population substructuring that might be obscured by deeper, hierarchical splits. For these nested STRUCTURE analyses, we removed one misidentified female from the *S. t. moreletti* group (see RAxML and full STRUCTURE results below); female *Sporophila* are often difficult to identify (Mason & Burns 2013; de Schauensee, 1952) and collectors occasionally misidentify females, such as this individual. We also removed loci that were not polymorphic or had more than 50% missing data within either population cluster. This generated a panel of 12510 loci and 44 individuals for the *S. t. morelleti* cluster and 12,912 loci and 19 individuals for the *S. t. torqueola* cluster.

We built species trees from biallelic SNPs with the SNAPP module (Bryant, Bouckaert, Felsenstein, Rosenberg, & RoyChoudhury, 2012) run with the program BEAST v2 (Bouckaert et al., 2014) to examine empirical support for different species delimitation scenarios (Leache, Fujita, Minin, & Bouckaert, 2014). Specifically, we compared the Bayes factors of marginal likelihood scores associated with three plausible species delimitation hypotheses (Grummer, Bryson, & Reeder, 2014). We subsampled the five individuals with the least amount of missing data from *S. t. torqueola* and *S. t. morelleti* in combination with the three *S. t. sharpei* and four outgroup samples. We restricted our data set to biallelic loci with a minor allele frequency >0.1 and <10% missing data; we then extracted 500 random loci from this subset. To acquire marginal likelihood scores, we performed path sampling with 48 steps. Each step included 100,000 generations in its MCMC chain with 10,000 pre‐burn‐in steps and 10% of subsequent generations removed as burn‐in; these settings generated MCMC chains that attained stationarity upon examining the posterior with Tracer (Rambaut, Suchard, Xie, & Drummond, 2014).

Finally, we estimated demographic parameters, including effective population size and migration rates, between subspecies groups in the *S. torqueola* species complex with the Bayesian program G‐PhoCS (Gronau, Hubisz, Gulko, Danko, & Siepel, 2011). Specifically, we extracted 1,000 randomly selected loci and included all individuals except for a single female that we suspect was misidentified (see RAxML and STRUCTURE results). We ran 2,000,000 iterations, remove 10% as burn‐in, and examined the posterior with Tracer (Rambaut et al., 2014). We ran 10 separate chains with different starting seeds to examine consistency among MCMC runs. We assumed an exponential distribution with a mean of 0.0001 for mutation‐scaled divergence time estimates (τ), an exponential distribution with mean of 0.01 for population size parameters (θ), and a Gamma priors (α = 0.002, β = 0.00001) for migration parameters (*m*). We calculated the effective population size (*N*_*e*_) in terms of θ = 4*N*_*e*_μ We used a mutation rate of 2.2 × 10^−9^ that is commonly applied to nuclear loci among vertebrate taxa (Kumar & Subramanian, 2002) to provide a rough estimate of *N*_*e*_ for populations in our study. While mutation rates vary substantially among regions of the genome and different taxa, we can compare relative differences in demographic parameters among populations considered in this study. We also calculated the proportion of individuals in population *x* that arrived by migration from population *y* per generation ($m_{\mathrm{yx}}\;\times \;\frac{\theta_{x}}{4}=M_{\mathrm{yx}}$; Gronau et al. 2011).

### 2.5 Biogeographic analyses

After generating alignments of mtDNA sequences of all available cyt b sequences of species in the genus *Sporophila*, we identified the optimal codon‐based partitioning scheme and corresponding models of nucleotide evolution for our mtDNA alignment using PartitionFinder (Guindon et al., 2010; Lanfear, Calcott, Ho, & Guindon, 2012; Lanfear, Frandsen, Wright, Senfeld, & Calcott, 2016). The best‐fit model included a separate partition for each codon position. Under the best‐fit model, the first codon position evolved under HKY + I + Γ, the second codon position evolved under GTR + I + Γ, and the third codon position evolved under a separate GTR + I + Γ model. We used BEAST 2 (Bouckaert et al., 2014) to infer an ultrametric phylogeny with our partitioned mtDNA alignment. We implemented a relaxed log‐normal clock with a substation rate of 2.1% per million years linked across partitions (Weir & Schluter, 2008). We ran three replicate BEAST 2 analyses for a total of 100 × 10^6^ generations and discarded the first 10 × 10^6^ generations as burn‐in. We assessed convergence across BEAST runs by ensuring that effective sample sizes surpassed 300 and that independent runs were congruent in topology. We combined the three runs and inferred a maximum clade credibility tree from the combined posterior distributions for biogeographic analyses.

We designated eight biogeographic regions to reconstruct the biogeographic history of *Sporophila* (from northwest to southeast; Figure 4b)*:* (1) northwest of the Isthmus of Tehuantepec, (2) Central American lowlands, (3) arid coastal South America, (4) northern savannas (including the Llanos and the Guianan savanna), (5) Amazon Basin forest clearings and scrub, (6) southern savannas (which includes the Chaco and Caatinga regions), and (7) Atlantic Forest clearings and scrub. To designate these regions, we compared shape files of species’ range maps (downloaded from http://birdlife.org) to calculate the amount of overlap between each species’ distribution shapefile and vectors corresponding to terrestrial ecoregions (Olson et al., 2001). We also included written descriptions of species’ preferred habitats and geographic distributions that we used to further refine the boundaries of our biogeographic regions and determine which regions each extant species occupies.

We subsequently modeled the evolution of geographic distributions within *Sporophila* using the BioGeoBEARS methodological framework (Matzke, 2014). In brief, BioGeoBEARS reconstructs biogeographic history by inferring ancestral geographic ranges using biogeographic areas delimited by users, a phylogeny, and an array of discrete Markov models with varying free parameters and assumptions. BioGeoBEARS users can alter settings to initialize and optimize Bayesian chains or maximum likelihood searches that explore different parameter space and subsequently compare the performance of models that differ in how species’ ranges evolve, such as the Dispersal‐Extinction‐Cladogenesis model (DEC; Ree & Smith, 2008) and a modified DEC model that includes an extra parameter to account for founder‐event speciation (DEC + J; Matzke, 2014). Users can then select the best‐performing model(s) via AICc scores and make inferences about the geographic ranges of ancestral nodes as well as the quantity and timing of different biogeographic events. Here, we used BioGeoBEARS to estimate ancestral ranges within *Sporophila* and determine how many times certain dispersal events—such as dispersal across the Isthmus of Tehuantepec—have occurred. We confirmed that our phylogeny had no extremely short branches, which are problematic for BioGeoBEARS, and set our initial parameter estimates to *d* = 0.0615, *e* = 0.0342, and *j* = 0.0001. All other settings were left at their default values. With eight biogeographic areas, we had a total of 128 possible states for each ancestral node as a node or species can occupy multiple areas. Following the maximum likelihood search, we displayed the state with the highest marginal probability for each node and the corresponding marginal probability.

## 3 RESULTS

### 3.1 Morphological differentiation within *Sporophila torqueola*

**Figure 2:**
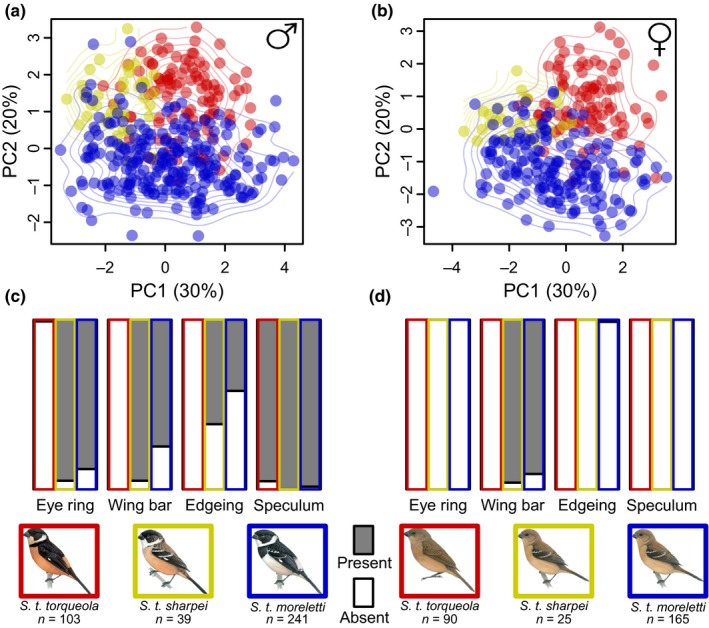
Morphometric analyses of three subspecies within the *Sporophila torqueola* species complex. Scatterplot of principal component axis 1 and principal component axis 2 for males (a) and females (b). Contour lines show the interpolated bivariate kernel density for each subspecies. Barplots showing frequency of plumage characters for males (c) and females (d). Colors that correspond to each subspecies and the presence or absence of a plumage character are shown at the key in the bottom along with sample sizes for each sex and subspecies

We found appreciable clustering corresponding to different subspecies groups within *S. torqueola* by comparing morphological measurements of over 600 specimens (Figure 2; Figures S1 and S2). After performing a principal component analysis using the seven morphological characters we measured, we found that PC1 and PC2 accounted for approximately 30% and 20% of the total variation, respectively (see Table S2 for loadings). The multinomial logistic regression with continuous morphological measurements and presence–absence plumage data measurements classified individuals to the correct subspecies (following on collectors’ classifications presumably based on geographic location and coarse phenotype) 90.3% of the time (*S. t. morelleti* 91.7% correctly classified; *S. t. sharpei* 59.0%; *S. t. torqueola* 99.0%), suggesting these groups are largely diagnosable.

### 3.2 UCE sequencing and population genetics

We recovered 4,376 UCE loci, which were 320 bp long on average (Figure S3). A total of 788 loci were invariant, while the remaining loci contained between 1 and 28 SNPs for a total of 17,130 SNPs (Figure S3). Variable loci contained between 0 and 25 parsimony informative sites (mean = 2.67 ± 3.21 SD per locus).

**Figure 3:**
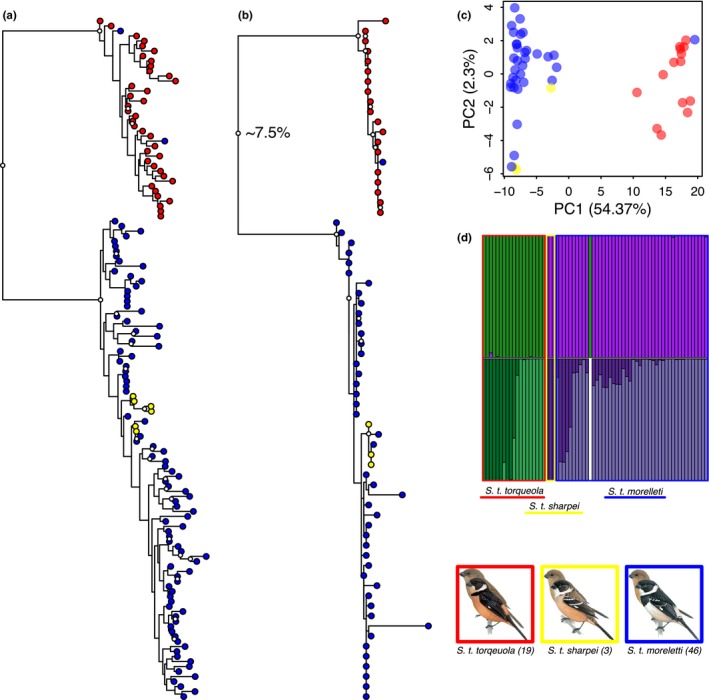
Phylogenetic and population genetic analyses of *S. torequola* with outgroup removed. (a) RAxML phylogeny built with 1,000 loci with highest number of parsimonious sites. Built by searching for the best tree and performing rapid bootstrapping in the same run (‐f a setting). Nodes with white circles have bootstrap support above 70. Tip colors correspond to taxa in the lower right corner. (b) RAxML phylogeny of cyt *b* mtDNA sequences. Built by searching for the best tree and performing rapid bootstrapping in the same run (‐f a setting). Nodes with white circles have bootstrap support above 70. Tip colors correspond to taxa in the lower right corner. (c) PCA plot constructed by filtering data set to include individuals with <85% missing data (*n* = 50) and loci with <75% missing data (*n* = 4,067). Dot colors correspond to taxa in the lower right corner. (d) STRUCTURE plot with optimal *K* value (2) determined by the Evanno method. Individuals are sorted according to taxa and by decreasing latitude within taxa, with rectangular boundary colors corresponding to taxa in the lower right corner. (e) STRUCTURE output for hierarchical analyses (optimal *K* = 2 for both) performed by subsetting the data set for each of the clusters identified and removing mismatched individuals in panel (d)

Our phylogeny of UCE loci constructed with RAxML recovered two deeply divergent lineages: one corresponding to vouchered specimens identified as *S. t. torqueola* and another that contained samples of *S. t. morelleti* and *S. t. sharpei* (Figure 3a). However, one individual female was mismatched; this sample was originally identified as *S. t. morelleti*, but was part of the clade that contained mostly *S. t. torqueola* samples. We recovered a similar topology and level of divergence in our phylogeny based on cyt b mitochondrial sequences (Figure 3b). Thus, phylogenetic reconstructions were highly congruent between nuclear and mitochondrial loci. The PCA plot similarly separated two groups along the first PCA axis, which accounted for 54.37% of the total variation (Figure 3c). Again, one individual was mismatched between their a priori subspecies designation and the population cluster that they were assigned to. We identified *K* = 2 as the optimal value for the number of STRUCTURE population clusters with outgroup sequences removed (Table 1). Using these settings, STRUCTURE assigned all *S. t. torqueola* samples to one population and all *S. t. morelleti* and *S. t. sharpei* samples to the other population, with the exception of the mismatched individual (Figure 3d). When we ran STRUCTURE on each of these clusters separately, we identified *K* = 2 as the optimal number of clusters for both subsets, with individuals assigned to either cluster based largely on latitude, suggesting a pattern of isolation by distance within both lineages (Figure 3e; Tables S4 and S5). Analyses that included outgroup sequences were congruent with analyses that did not include outgroup samples (Figure S4 and Table S3). Bayes factor species delimitation strongly supported a scenario with *S. t. torqueola* split from *S. t. morelleti* with Bayes factors >10 considered decisive (Table 2; Kass & Raftery, 1995). Our demographic analyses with G‐PhoCS revealed very low levels of gene flow between *S. t. morelleti*,* S. t. torqueola*, and *S. t. minuta* (Table 3).

**Table 1:** Output from STRUCTURE analyses to determine the optimal number of population clusters based on patterns in log‐likelihood scores for 68 ingroup individuals

*K*	Reps	Mean LnP(*K*)	Stdev LnP(*K*)	Ln’(*K*)	|Ln’’(*K*)|	Delta *K*
1	10	−259379.15	6.99	NA	NA	NA
**2**	**10**	−**128340.44**	**11.66**	**131038.71**	**135559.23**	**11622.95**
3	10	−132860.96	56.82	−4520.52	67109.51	1181.05
4	10	−204490.99	152532.90	−71630.03	140483.79	0.92
5	10	−135637.23	7996.12	68853.76	1248863.56	156.18
6	10	−1315647.03	576353.39	−1180009.80	NA	NA

The favored run has the highest Delta *K* score, in which higher scores indicate greater changes in likelihood scores and less standard deviation in likelihood scores among replicate runs for a given *K* value. The row corresponding to the *K* value with the highest Delta *K* scores is shown in bold.

**Table 2:** Empirical results for Bayes Factor Species delimitation in the *Sporophila torqueola* complex

Model	Species	ML	Rank	BayesFactor
Current taxonomy	1	−2824.46	2	–
**Recognize ** ***S. t. torqueola and S. t. moreletti***	**2**	−**716.39**	**1**	−**4216.14**
Recognize *S. t. torqueola, S. t. moreletti, and S. t. sharpei*	3	−5133.31	3	4617.7

ML stands for the marginal likelihood (log_*e*_). Bayes factor is calculated with the equation BF = 2 × (ML_CurrentTaxonomy _− ML_AlternativeModel_). A negative BayesFactor value indicates support for an alternative model over the current taxonomy. The favored species delimitation model is shown in bold. A difference in ≥10 of BayesFactors indicates strong support for an alternative model; our results therefore overwhelmingly support a model of two species that recognizes both *S. t. torqueola* and *S. t. moreletti/sharpei* as separate species.

**Table 3:** Estimates of demographic parameters based on 1,000 ultraconserved element loci using the program G‐PhoCS for the *Sporophila torqueola* species complex

*N*_*e*_		*M*	
Taxon	Mean *N*_*e*_ (HPD)	Migration band	Mean *m* (HPD)
*Sharpei*	12612.51 (7954.55–30681.82)	*m*_*sharpei ‐> morelleti*_	3.80e‐1 (4.03e‐2–7.78e‐1)
*Morelleti*	271931.72 (229545.46–312500.0)	*m*_*morelleti ‐> sharpei*_	5.84e‐3 (5.55e‐11–4.66e‐2)
*Torqueola*	69637.19 (52272.73–75000.00)	*m*_*morelleti + sharpei* ‐> *torqueola*_	2.11e‐2 (7.23e‐8–2.94e‐2)
*Sharpei* + *Morelleti*	101862.56 (17045.45–117045.45)	*m*_*torqueola* ‐> *morelleti + sharpei*_	1.76e‐2 (2.08e‐3–2.52e‐2)
Root	116044.19 (39772.73–340909.09)		

Effective population sizes (*N*_*e*_) were calculated assuming a mutation rate of 2.2 × 10^−9^. Migration bands are shown in effective number of migrants per generation. The 95% lower and higher highest probability density (HPD) values represent the predictive distribution of parameter estimates and are analogous to confidence intervals and are shown in parentheses next to each mean value.

### 3.3 Biogeography of *Sporophila torqueola*

**Figure 4:**
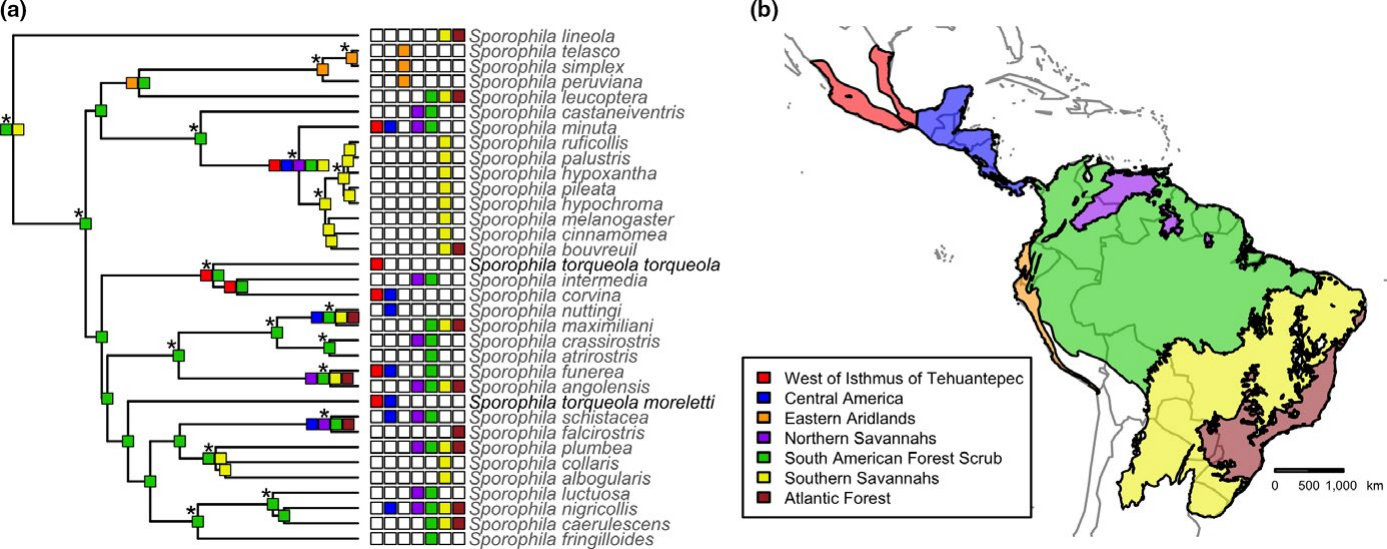
(a) Inferred phylogeny of the genus *Sporophila* estimation of the probabilities of ancestral ranges via the best‐fit model in BioGeoBEARS (DEC model). Asterisks indicate strongly supported nodes with over 95 posterior probability. The most likely ancestral range is indicated for each node, while the range for each extant species is shown at the tips of the phylogeny. The ancestral range of *Sporophila* was most likely forest scrub in central South America, and multiple lineages have crossed the Isthmus of Tehuantepec, including two lineages that are currently considered conspecific (*S. torqueola torqueola* and *S. torqueola morelleti* shown in bold). (b) Biogeographic regions used in the BioGeoBEARS analyses conducted in this study. The colors corresponding to the key in the lower left represent the same biogeographic regions in panel (a)

We inferred a mitochondrial phylogeny that was concordant with previous phylogenies of the group (Burns et al., 2014; Mason & Burns, 2013). Our mtDNA sequences corresponding to *S. t. torqueola* and *S. t. morelleti* were not inferred as each other's closest relatives. Rather, we inferred that *S. t. torqueola* is sister to a mainly Mesoamerica‐northern South America clade containing *S. intermedia* and *S. corvina* with strong nodal support, while *S. t. moreletii* and *S. t. sharpei* were more closely related to a clade comprised of other species of *Sporophila* from Central and South America (e.g., *S. schistacea, S. plumbea*; Figure 4). After comparing the likelihood of our data given two biogeographic models, we found that a DEC model (AIC_WT_ = 0.73) was favored over a DEC + J model (AIC_WT_ = 0.27). Using the favored DEC model, we calculated the marginal probabilities for each of the 128 possible states at each node. Many nodes had high marginal probabilities for specific states (Figure 4a); deeper nodes, however, typically had more uncertainty in estimated ancestral ranges (Figure S6). The node corresponding to the most recent common ancestor of all *Sporophila* was estimated to occupy clearings and scrub of the Amazon Basin and southern Savannas in South America, albeit with low certainty (.09 probability). Similarly, the most likely estimated ranges of the majority of deeper nodes in *Sporophila* included clearings and scrub of the Amazon Basin, suggesting that *Sporophila* originated in South America. While it remains unclear which biogeographic areas the ancestral nodes in *Sporophila* occupied, it seems certain that there have been multiple dispersal events out of South America into Central America, and across the Isthmus of Tehuantepec—including two separate isthmus crossings that correspond to *S. t. torqueola* and *S. t. morelleti* (Figure 4).

## 4 DISCUSSION

Analyses of our large panel of nuclear markers developed from UCE loci demonstrated that *S. t. torqueola* of the Pacific coast of Mexico is strongly differentiated from *S. t. morelleti* and *S. t. sharpei* (Figure 3). This pattern is corroborated by mitochondrial DNA, which further suggested that *S. t. torqueola* is more closely related to other species within *Sporophila* than *S. t. morelleti* or *S. t. sharpei* (Figure 4). Thus, although the deeply divergent lineages recovered here are diagnosable in morphology and plumage characters (Figure 2), similarity in overall plumage patterning has heretofore obscured the evolutionary history among lineages within the previously synonymized *S. torqueola* species complex. These findings suggest a revised biogeographic history for the genus *Sporophila* that includes multiple crossings of the Isthmus of Tehuantepec (Figure 4) and highlight the Pacific coast of Mexico as an important region of endemism (Arbeláez‐Cortés, Milá, & Navarro‐Sigüenza, 2014; Arbeláez‐Cortés, Roldán‐Piña, & Navarro‐Sigüenza, 2014; García‐Trejo & Navarro‐Sigüenza, 2004; Peterson & Navarro‐Sigüenza, 2000). Below, we discuss the implications of these results toward the broader conceptual goal of understanding the evolutionary processes that underlie avian biodiversity in the Neotropics.

### 4.1 Diversification of the *Sporophila torqueola* Complex

Our analysis of phenotypic variation within the *S. torqueola* complex revealed largely diagnosable clusters of morphological variation and plumage characters that correspond to currently recognized subspecies (Figure 2). For the purpose of this discussion and clarity, we hereafter refer to these lineages as separate species: *S. torqueola* and *S. morelleti*. Given that multiple plumage and morphological characters differ between the western *S. torqueola* and the eastern *S. morelleti*, there are presumably multiple regions of the genome underlying phenotypic differentiation in this system (Mackay, Stone, & Ayroles, 2009; Stranger, Stahl, & Raj, 2011). Our genetic analyses corroborate this assertion: The phenotypically differentiated *S. torqueola* expresses strong genetic differentiation from *S. morelleti* among many unlinked loci (Figure 4). Thus, genetic differentiation is prevalent throughout the genome, which differs from a suite of recent studies that have found patterns of widespread genomic homogeneity with a small number of loci likely underlying phenotypic differentiation in other avian taxa (Mason & Taylor, 2015; Poelstra et al., 2014; Toews, Taylor, et al., 2016). Indeed, the deep evolutionary split that we documented between *S. torqueola* and *S. morelleti* is in stark contrast to the shallow evolutionary history that characterizes the southern capuchino radiation in the same genus, in which rapid and recent phenotypic differentiation and speciation are driven by a few loci of large effect (Campagna et al., 2017).

We recovered reciprocal monophyly between *S. torqueola* and *S. morelleti* with the exception of a mismatched individual (Figure 3a,b). This individual was a female (voucher UWBM 112783) identified as *S. morelleti* based on where it was collected in the Campeche state of the Yucatan peninsula (Table S2), yet our phylogenetic analyses reveal this individual was likely misidentified. Given the proximity of the Campeche region to the putative contact zone between *S. torqueola* and *S. morelleti,* this female may either represent a recent, natural dispersal event or could be an escaped caged bird (Alves & de Faria Lopes, 2016; Carrete & Tella, 2008; CONABIO 1996; do Nascimento & Czaban, 2015; Aguilar Rodriguez, 1992). We do not see any evidence of introgression in either lineage, however, as indicated by the lack of admixture for this individual and all other samples included in the STRUCTURE analysis (Figure 3e). This finding highlights the challenges of identifying female *Sporophila*, which are very similar in overall appearance (de Schauensee, 1952), and warrants caution in interpreting species identifications of female *Sporophila* based on phenotypes and presumed geographic distributions alone.

The deep molecular divergence and polyphyly that we recovered between *S. torqueola* and *S. morelleti* have been obscured by broad‐scale similarity in plumage patterning and geographic distributions. Convergent phenotypes and biogeographic affinities have similarly contradicted phylogenetic relationships in numerous taxa, such as sponges (Schuster et al., 2015), wasps (Ceccarelli & Zaldívar‐Riverón, 2013), gobies (Ellingson, Swift, Findley, & Jacobs, 2014), sierra finches (Campagna et al., 2011)*,* and antbirds (Thamnophilidae; Bravo, Remsen, & Brumfield, 2014), among many others (Funk & Omland, 2003). Our study therefore contributes to an ever‐growing knowledge of evolutionary relationships among extant taxa and patterns of phenotypic evolution. We presented multiple lines of evidence that support an integrative taxonomic revision to recognize *S. torqueola* and *S. morelleti* as separate species based on phenotypic differentiation, low estimates of gene flow in demographic models, and multispecies coalescent species delimitation (Fujita, Leaché, Burbrink, Mcguire, & Moritz, 2012). The genus *Sporophila* has a remarkably high speciation rate that is similar to Darwin's Finches (Burns et al., 2014; Campagna et al., 2017). Our study joins others in describing heretofore unrecognized species‐level diversity in *Sporophila* (but see Navarro‐Sigüenza & Peterson, 2004; Rising, 2017), such as the newly recognized species *S. beltoni* (Repenning & Fontana, 2013). Thus, rapid diversification seems to characterize the genus *Sporophila*, suggesting that future studies on widespread, phenotypically variable congeners, such as *S. minuta* and *S. corvina*, may reveal even more species‐level biodiversity that is currently unrecognized. Our improved knowledge of species‐level diversity and phylogenetic relationships within *Sporophila* sheds new light on the biogeography of the genus and add to our understanding of diversification among Mesoamerican biota.

### 4.2 Biogeography of Neotropical seedeaters

The deep molecular divergence and phenotypic differentiation that we recovered within the *S. torqueola* complex changes our biogeographic perspective of diversification in Neotropical seedeaters (Figure 4). Our BioGeoBEARS analysis suggested that many of the deeper nodes occupied clearings and scrub in the Amazon basin, although there is a lot of uncertainty in which exact states the deepest ancestral nodes in *Sporophila* occupied (Figure S6). Extinction raises further uncertainty in inferring ancestral states deep in evolutionary time with confidence (Crisp, Trewick, & Cook, 2011). Thus, while the exact ancestral range of the most recent common ancestor of all *Sporophila* remains unknown, our analysis suggests a biogeographic origin in South America.

We inferred repeated dispersal out of South America into Mesoamerica, and across the Isthmus of Tehuantepec (Figure 4). This repeated dispersal is nested within a deeper biogeographic history of the nine‐primaried oscines, which entered the Americas through Arctic Beringia and exchanged continental biota before and after the closure of the Isthmus of Panama (Barker, Burns, Klicka, Lanyon, & Lovette, 2015; Smith & Klicka, 2010). The Isthmus of Tehuantepec is a narrow continental area separating the Gulf Coast and the Pacific Ocean that is the product of a complex geologic history of tectonic uplift and sea level change over the past 6 million years since the late Miocene (Barrier, Velasquillo, Chavez, & Gaulon, 1998; Ferrusquía‐Villafranca, 1993). Periodic episodes of geographic isolation imposed by uplift of the Isthmus of Tehuantepec and glacial oscillations in sea level have served as important biogeographic events for other taxa, including freshwater fishes (Huidobro, Morrone, Villalobos, & Alvarez, 2006), insects (Halffter, 1987), snakes (Bryson, García‐Vázquez, & Riddle, 2011; Castoe et al., 2009; Daza, Castoe, & Parkinson, 2010), shrews (Esteva, Cervantes, Brant, & Cook, 2010), and other birds (Barber & Klicka, 2010; Cortés‐Rodríguez et al., 2013). The distributional patterns observed in *S. torqueola* and *S. morelleti* are similar to other Neotropical taxa that span the coastal lowlands below 1,000 m and border the Mexican mountain systems (Arbeláez‐Cortés & Navarro‐Sigüenza, 2013; Huidobro et al., 2006; Lavinia et al., 2015), which roughly corresponds to the Neotropical region of the established transition zones between Neotropical and Nearctic fauna in Mexico (Halffter & Morrone, 2017; Morrone, 2010). Changes in sea level and the complex orogeny of the Mexican mountains may have therefore contributed to the contemporary distribution of *S. morelleti* and *S. torqueola* by restricting them to glacial refugia on the coastal plains of the Atlantic and Pacific Coasts, respectively. Today, the Isthmus of Tehuantepec and the lowlands to the southeast of the Sierra Madre del Sur mountains act as a potential “node,” or connection point, between the western *S. torqueola* group and the eastern *S. morelleti* group; yet we recovered no evidence of gene flow between these two lineages. If we accept that there is little ongoing gene flow between *S. torqueola* and *S. moreletti* (Table 3), then our study adds to frequently unrecognized species‐level endemism in the Pacific coastal plains of Mexico.

The Pacific coastal plains of Mexico, which are bounded by the Sierra Madre Occidental and Sierra Madre del Sur, are home to numerous endemics. Over 20 endemic birds occur along the Pacific coast of Mexico (Escalante, Navarro‐Sigüenza, & Peterson, 1993), alongside more than 30 endemic amphibians (García, 2006), over 120 endemic nonavian reptiles (García, 2006), numerous mammals (Escalante, Szumik, & Morrone, 2009; García‐Trejo & Navarro‐Sigüenza, 2004), and terrestrial invertebrates (Morrone & Márquez, 2001). This region comprises the northwestern limits of the Neotropical lowlands that characterize the Mexican transition zone (Halffter & Morrone, 2017). The striking ecological gradients that characterize elevational rise in the Sierra Madre Occidental combined with periodic isolation from the Atlantic coastal plains via rises in sea level and submersion of the Isthmus of Tehuantepec may have contributed to ecological specialization, biogeographic isolation, and endemism in the region. Elevating *S. t. torqueola* to species status, which we recommend based on our findings here, further contributes to understanding endemism in the Pacific coastal plains of Mexico, highlighting this area as a biodiversity hotspot worthy of conservation efforts in Mesoamerica (García‐Trejo & Navarro‐Sigüenza, 2004; Peterson & Navarro‐Sigüenza, 2000).

## 5 CONCLUSION

In summary, our study documents deep molecular divergence between phenotypically differentiated subspecies that have heretofore been considered a single species of Neotropical bird. The polyphyly that we recover within the *S. torqueola* species complex emphasizes the capacity of phenotypic convergence and geographic affinities to obscure true evolutionary relationships. Our improved phylogeny of the genus *Sporophila* transforms our understanding of the biogeographic history of this lineage by suggesting there have been multiple, repeated dispersal events out of South America into Mesoamerica. Finally, our study suggests a taxonomic split to recognize *S. morelleti* and *S. torqueola* as separate species, which exhibit pronounced phenotypic and genetic differentiation and are not sister species, highlighting the Pacific coast and lowlands of Mexico as a region that harbors high, yet underrecognized, levels of endemism.

## DATA ACCESSIBILITY

Raw Illumina reads are available via the NCBI Short Read Archive. A genepop file of SNPs used in the analyses presented here is available through Dryad. Mitochondrial gene sequences are available through GenBank. Metadata for molecular analyses, including voucher information, sampling localities are available through Dryad. Morphological data, including voucher information and raw measurements, are on Dryad. R scripts used to run biogeographic analyses, and generate figures are uploaded to Dryad.

## CONFLICT OF INTEREST

None declared.

## AUTHOR CONTRIBUTIONS

N.A.M., A.O.V., I.J.L., and A.G.N.S. conceived the project. N.A.M. and A.O.V. carried out the laboratory work. A.O.V. measured morphological and plumage characters from specimens. N.A.M. developed the bioinformatics pipeline and analyzed the molecular data. N.A.M., A.O.V., I.J.L., and A.G.N.S. wrote and edited the manuscript.
