# Supplementary material for: Hidden endemism, deep polyphyly, and repeated dispersal across the Isthmus of Tehuantepec: Diversification of the White‐collared Seedeater complex (Thraupidae: Sporophila torqueola)
Source: Ecol Evol. 2018 Jan 12;8(3):1867–81. doi: 10.1002/ece3.3799 (PMC5792519; doi:10.1002/ece3.3799)

**Hidden endemism, deep polyphyly, and repeated dispersal across the Isthmus of Tehuantepec: diversification of the White-collared Seedeater complex (Thraupidae: *Sporophila torqueola*)**

Nicholas A. Mason, Arturo Olvera-Vital, Irby J. Lovette, Adolfo Navarro-Sigüenza

SUPPLEMENTARY MATERIAL

Contents:

Supplementary Table S1: Voucher information for samples used in genetic analyses.

Supplementary Table S2: Loadings for first two principal component axes of morphological measurements.

Supplementary Table S3: STRUCTURE analyses output for ingroup and outgroup taxa.

Supplementary Table S4: STRUCTURE analyses output for *S. t. torqueola* subset.

Supplementary Table S5: STRUCTURE analyses output for *S. t. moreletti* subset.

Supplementary Figure S1: Boxplots of male morphometric data.

Supplementary Figure S2: Boxplots of female morphometric data.

Supplementary Figure S3: Summary of UCE assembly and SNP calling statistics.

Supplementary Figure S4: Population genetic analyses of full data set.

Supplementary Figure S5: Phylogeny of mitochondrial DNA of focal individuals and related taxa

Supplementary Figure S6: BioGeoBEARS probabilities for the estimated ancestral range of each node.

Supplementary Table S1: Vouchered specimens used in genetic analyses. Collection codes are as follows: LSU: Louisiana State University Museum of Natural Science; UWBM: University of Washington Burke Museum; CUMV: Cornell University Museum of Vertebrates; MZFC: El Museo de Zoología "Alfonso L. Herrera". Samples that have “NA” in the locality field lack a specific written locality beyond the information displayed below.

| Species | Subspecies | Collection | Catalog number | Country | State / Province | Locality | Latitude | Longitude |
| --- | --- | --- | --- | --- | --- | --- | --- | --- |
| *S. torqueola* | *moreletti* | LSU | 60718 | Honduras | Atlántida | NA | 15.666667 | -87 |
| *S. torqueola* | *moreletti* | LSU | 60719 | Honduras | Atlántida | NA | 15.666667 | -87 |
| *S. torqueola* | *moreletti* | UWBM | 100783 | Mexico | Veracruz | Montepio | 18.16995 | -95.09344 |
| *S. torqueola* | *moreletti* | UWBM | 100784 | Mexico | Veracruz | Montepio | 18.16995 | -95.09344 |
| *S. torqueola* | *moreletti* | UWBM | 100899 | Mexico | Veracruz | Balzapote, Finca la Iguana | 18.62188 | -95.0729 |
| *S. torqueola* | *moreletti* | UWBM | 101342 | Mexico | Campeche | Escarcega, 4 km N | 18.6413 | -90.6754 |
| *S. torqueola* | *moreletti* | UWBM | 101350 | Mexico | Campeche | Escarcega, 4 km N | 18.6413 | -90.6754 |
| *S. torqueola* | *moreletti* | UWBM | 101371 | Mexico | Campeche | Escarcega, 4 km N | 18.6413 | -90.6754 |
| *S. torqueola* | *moreletti* | UWBM | 104302 | Guatemala | Retalhuleu | San Felipe Retalhuleu, 5 km S; Finca El Nino | 14.6 | -91.611667 |
| *S. torqueola* | *moreletti* | UWBM | 105926 | Guatemala | Retalhuleu | San Felipe Retalhuleu, 5 km S; Finca El Nino | 14.6 | -91.611667 |
| *S. torqueola* | *moreletti* | UWBM | 105927 | Guatemala | Retalhuleu | San Felipe Retalhuleu, 5 km S; Finca El Nino | 14.6 | -91.611667 |
| *S. torqueola* | *moreletti* | UWBM | 94253 | Honduras | Copán | Copán Ruinas, 10 km ENE | 14.866667 | -89.05 |
| *S. torqueola* | *moreletti* | UWBM | 94254 | Honduras | Copán | Copán Ruinas, 10 km ENE | 14.866667 | -89.05 |
| *S. torqueola* | *moreletti* | CUMV | 10023 | Mexico | Veracruz | Jalapa | 19.533982 | -96.9169327 |
| *S. torqueola* | *moreletti* | CUMV | 18590 | Costa Rica | Cartago | NA | 9.800082 | -83.661518 |
| *S. torqueola* | *moreletti* | CUMV | 18591 | Costa Rica | Cartago | NA | 9.800082 | -83.661518 |
| *S. torqueola* | *moreletti* | CUMV | 36352 | Nicaragua | Leon | NA | 12.578834 | -86.578796 |
| *S. torqueola* | *moreletti* | CUMV | 36353 | Nicaragua | Leon | NA | 12.578834 | -86.578796 |
| *S. torqueola* | *moreletti* | UWBM | 100687 | Mexico | Campeche | Escarcega, 4 km N | 18.6413 | -90.6754 |
| *S. torqueola* | *moreletti* | UWBM | 100827 | Mexico | Veracruz | Montepio | 18.63995 | -95.09344 |
| *S. torqueola* | *moreletti* | UWBM | 100828 | Mexico | Veracruz | Montepio | 18.16995 | -95.09344 |
| *S. torqueola* | *moreletti* | UWBM | 100829 | Mexico | Veracruz | Montepio | 18.63995 | -95.09344 |
| *S. torqueola* | *moreletti* | UWBM | 100830 | Mexico | Veracruz | Montepio | 18.16995 | -95.09344 |
| *S. torqueola* | *moreletti* | UWBM | 100831 | Mexico | Veracruz | Montepio | 18.63995 | -95.09344 |
| *S. torqueola* | *moreletti* | UWBM | 101326 | Mexico | Campeche | Escarcega, 4 km N | 18.6413 | -90.6754 |
| *S. torqueola* | *moreletti* | UWBM | 104298 | Guatemala | Retalhuleu | San Felipe Retalhuleu, 5 km S; Finca El Nino | 14.6 | -91.611667 |
| *S. torqueola* | *moreletti* | UWBM | 105997 | Guatemala | Retalhuleu | San Felipe Retalhuleu, 5 km S; Finca El Nino | 14.6 | -91.611667 |
| *S. torqueola* | *moreletti* | UWBM | 105998 | Guatemala | Retalhuleu | San Felipe Retalhuleu, 5 km S; Finca El Nino | 14.6 | -91.611667 |
| *S. torqueola* | *moreletti* | UWBM | 112783 | Mexico | Campeche | Escarcega, 4 km N | 18.6413 | -90.6754 |
| *S. torqueola* | *moreletti* | UWBM | 94258 | Honduras | Copán | Copán Ruinas, 10 km ENE | 14.866667 | -89.05 |
| *S. torqueola* | *moreletti* | UWBM | 94272 | Honduras | Copán | Copán Ruinas, 10 km ENE | 14.866667 | -89.05 |
| *S. torqueola* | *moreletti* | UWBM | 94273 | Honduras | Copán | Copán Ruinas, 10 km ENE | 14.866667 | -89.05 |
| *S. torqueola* | *moreletti* | MZFC | 17053 | Mexico | Chiapas | Pijijiapan, Rancho Nueva Ensenada, Col. Miguel Alemán Valdés | 15.560944 | -93.0168611 |
| *S. torqueola* | *moreletti* | MZFC | 23124 | Mexico | Chiapas | Camino a Zona Arqueológica de Toniná, Rancho los Abuelos | 16.8874 | -92.0206 |
| *S. torqueola* | *moreletti* | MZFC | 25874 | Mexico | Chiapas | Reserva El Silencio | 14.67249 | -92.23057 |
| *S. torqueola* | *moreletti* | MZFC | 25885 | Mexico | Chiapas | Reserva El Silencio | 14.67249 | -92.23057 |
| *S. torqueola* | *moreletti* | MZFC | 11971 | Mexico | Oaxaca | La Cabaña a 6.2 km NO de San Francisco La Paz | 17.066819 | -94.05 |
| *S. torqueola* | *moreletti* | MZFC | 11558 | Mexico | Puebla | Cuitchat, 8 km NE Cuetzalan | 20.091666 | -97.5166666 |
| *S. torqueola* | *moreletti* | MZFC | 18295 | Mexico | Tabasco | Ejido Central Fournier (Segunda Sección), Laguna El Rosario | 17.870833 | -93.8833333 |
| *S. torqueola* | *moreletti* | MZFC | 18294 | Mexico | Tabasco | Ejido Central Fournier (Segunda Sección), Laguna El Rosario | 17.870833 | -93.8833333 |
| *S. torqueola* | *moreletti* | MZFC | 17578 | Mexico | Veracruz | Club de Pesca, Km 10 Carretera Zamora-Tecolutla | 20.475333 | -97.03018 |
| *S. torqueola* | *moreletti* | MZFC | 24216 | Mexico | Veracruz | San Felipe Cerro Quebrado, inicio de camino a Ignacio Zaragoza | 20.005083 | -96.9162777 |
| *S. torqueola* | *moreletti* | MZFC | 26030 | Mexico | Veracruz | Ejido La Perla de San Martín, Campamento B | 18.55125 | -95.1236666 |
| *S. torqueola* | *moreletti* | MZFC | 25763 | Mexico | Yucatan | Río Lagartos, Reserva de la Biosfera Ría Lagartos | 21.56292 | -88.07627 |
| *S. torqueola* | *moreletti* | UWBM | 94256 | Honduras | Copán | Copán Ruinas, 10 km ENE | 14.866667 | -89.05 |
| *S. torqueola* | *moreletti* | LSU | 60725 | Honduras | Atlántida | NA | 15.666667 | -87 |
| *S. torqueola* | *moreletti* | CUMV | 10490 | Mexico | Tamaulipas | Rio Sabinas, near Gomez Farias | 23.023812 | -99.0753156 |
| *S. torqueola* | *sharpei* | LSU | 43282 | USA | Texas | NA | 26.645134 | -99.174507 |
| *S. torqueola* | *sharpei* | LSU | 43283 | USA | Texas | NA | 26.645134 | -99.174507 |
| *S. torqueola* | *torqueola* | UWBM | 82394 | Mexico | Sinaloa | El Fuerte; Tehueco | 26.304 | -108.699 |
| *S. torqueola* | *torqueola* | UWBM | 82617 | Mexico | Sinaloa | El Fuerte; Tehueco | 26.304 | -108.699 |
| *S. torqueola* | *torqueola* | UWBM | 86547 | Mexico | Sinaloa | El Fuerte; Tehueco | 26.324 | -108.69 |
| *S. torqueola* | *torqueola* | UWBM | 88978 | Mexico | Sinaloa | Los Mochis Ejido; Las Lineas | 25.9647 | -108.9827 |
| *S. torqueola* | *torqueola* | UWBM | 90893 | Mexico | Sinaloa | El Fuerte; 5-6 km NW of Ejido Tesila, along main canal | 26.3005 | -108.8225 |
| *S. torqueola* | *torqueola* | UWBM | 82473 | Mexico | Sinaloa | El Fuerte; Boca del Arroyo | 26.3 | -108.77 |
| *S. torqueola* | *torqueola* | UWBM | 82618 | Mexico | Sinaloa | El Fuerte; Tehueco | 26.304 | -108.699 |
| *S. torqueola* | *torqueola* | UWBM | 88920 | Mexico | Sinaloa | El Fuerte; 5-6 km NW of Ejido Tesila, near main canal | 26.3005 | -108.8225 |
| *S. torqueola* | *torqueola* | UWBM | 90703 | Mexico | Sinaloa | El Fuerte; Ejido Tesila | 26.290016 | -108.78466 |
| *S. torqueola* | *torqueola* | MZFC | 27709 | Mexico | Guerrero | Papalutla Tenango | 18.029583 | -98.9159555 |
| *S. torqueola* | *torqueola* | MZFC | 20729 | Mexico | Guerrero | Los Cirilos | 17.645 | -101.466111 |
| *S. torqueola* | *torqueola* | MZFC | 24023 | Mexico | Guerrero | Lomas de Chapultepec | 16.79972 | -99.66933 |
| *S. torqueola* | *torqueola* | MZFC | 27712 | Mexico | Oaxaca | Parque Nacional Lagunas de Chacahua | 15.96683 | -97.6881 |
| *S. torqueola* | *torqueola* | MZFC | 19632 | Mexico | Sinaloa | Matatan 2 km W, El Limón junto al Rio Baluarte | 23.025 | -105.751666 |
| *S. torqueola* | *torqueola* | MZFC | 15842 | Mexico | Zacatecas | Rancho Chalchisco, 10 Km W de Jalpa | 21.632111 | -103.075786 |
| *S. torqueola* | *torqueola* | CUMV | BTCPM260 | Mexico | Oaxaca | Parque Nacional Lagunas de Chacahua | 15.96683 | -97.6881 |
| *S. torqueola* | *torqueola* | CUMV | BTCPM270 | Mexico | Oaxaca | Parque Nacional Lagunas de Chacahua | 15.96683 | -97.6881 |
| *S. torqueola* | *torqueola* | CUMV | BTNAM086 | Mexico | Oaxaca | Parque Nacional Lagunas de Chacahua | 15.96683 | -97.6881 |
| *S. torqueola* | *torqueola* | CUMV | BTNAM085 | Mexico | Oaxaca | Parque Nacional Lagunas de Chacahua | 15.96683 | -97.6881 |
| *Sporophila* | *minuta* | CUMV | BTCPM250 | Mexico | Oaxaca | Parque Nacional Lagunas de Chacahua | 15.96683 | -97.6881 |
| *Sporophila* | *minuta* | CUMV | BTCPM276 | Mexico | Oaxaca | Parque Nacional Lagunas de Chacahua | 15.96683 | -97.6881 |
| *Sporophila* | *minuta* | UWBM | 55986 | Nicaragua | Tipitapa | along shore of Lago de Managua, near Río Tipitapa | 12.204196 | -86.112334 |
| *Sporophila* | *minuta* | UWBM | 70241 | Nicaragua | Tipitapa | along shore of Lago de Managua, near Río Tipitapa | 12.204196 | -86.112334 |

Supplementary Table S2: Loadings for first two principal component axes of morphological measurements for males and females together. The percentage of the total variation that each axis accounts for is shown in parentheses in the top row.

|  | PC1  (30.05%) | PC2  (20.49%) |
| --- | --- | --- |
| Culmen length | 0.45 | -0.32 |
| Gonys length | 0.46 | -0.25 |
| Bill depth at base | 0.45 | -0.17 |
| Bill width at base | 0.33 | 0.06 |
| Tarsus length | 0.23 | 0.01 |
| Hallux length | 0.28 | -0.10 |
| Length of central rectrix | 0.26 | 0.64 |
| Wing chord length | 0.28 | 0.62 |

Supplementary Table S3: Output from STRUCTURE analyses to determine the optimal number of population clusters based on patterns in log likelihood scores for 68 ingroup individuals and four outgroup individuals. The favored run has the highest Delta K score, in which higher scores indicate greater changes in likelihood scores and less standard deviation in likelihood scores among replicate runs for a given K value. The row corresponding to the K value with the highest Delta K scores is shown in bold.

| K | Mean LnP(K) | Stdev LnP(K) | Ln'(K) | \|Ln''(K)\| | Delta K |
| --- | --- | --- | --- | --- | --- |
| 1 | -344633.23 | 3.85 | NA | NA | NA |
| 2 | -205208.27 | 3549.15 | 139424.96 | 70049.59 | 19.74 |
| **3** | **-135832.89** | **16.26** | **69375.38** | **81476.71** | **5009.41** |
| 4 | -147934.22 | 2241.10 | -12101.33 | 48740.88 | 21.75 |
| 5 | -208776.43 | 201130.92 | -60842.21 | 619579.94 | 3.08 |
| 6 | -889198.58 | 310249.47 | -680422.15 | NA | NA |

Supplementary Table S4: Output from STRUCTURE analyses to determine the optimal number of population clusters based on patterns in log likelihood scores for 19 *S. t. torqueola* individuals. The favored run has the highest Delta K score, in which higher scores indicate greater changes in likelihood scores and less standard deviation in likelihood scores among replicate runs for a given K value. The row corresponding to the K value with the highest Delta K scores is shown in bold.

| K | Mean LnP(K) | Stdev LnP(K) | Ln'(K) | \|Ln''(K)\| | Delta K |
| --- | --- | --- | --- | --- | --- |
| 1 | -23816.47 | 10.31 | NA | NA | NA |
| **2** | **-21836.76** | **5.21** | **1979.71** | **3858.05** | **740.32** |
| 3 | -23715.10 | 1257.11 | -1878.34 | 8984.36 | 7.15 |
| 4 | -34577.80 | 13289.22 | -10862.70 | 1714.17 | 0.13 |
| 5 | -47154.67 | 18966.95 | -12576.87 | 7623.12 | 0.40 |
| 6 | -67354.66 | 35080.51 | -20199.99 | 3058.12 | 0.09 |
| 7 | -90612.77 | 63561.66 | -23258.11 | 147.77 | 0.00 |
| 8 | -114018.65 | 59652.37 | -23405.88 | 2935.32 | 0.05 |
| 9 | -140359.85 | 82054.53 | -26341.20 | 17579.89 | 0.21 |
| 10 | -149121.16 | 99840.02 | -8761.31 | NA | NA |

Supplementary Table S5: Output from STRUCTURE analyses to determine the optimal number of population clusters based on patterns in log likelihood scores for 44 *S. t. moreletti* individuals. The favored run has the highest Delta K score, in which higher scores indicate greater changes in likelihood scores and less standard deviation in likelihood scores among replicate runs for a given K value. The row corresponding to the K value with the highest Delta K scores is shown in bold.

| K | Mean LnP(K) | Stdev LnP(K) | Ln'(K) | \|Ln''(K)\| | Delta K |
| --- | --- | --- | --- | --- | --- |
| 1 | -23816.47 | 10.31 | NA | NA | NA |
| **2** | **-21836.76** | **5.21** | **1979.71** | **3858.05** | **740.32** |
| 3 | -23715.10 | 1257.11 | -1878.34 | 8984.36 | 7.15 |
| 4 | -34577.80 | 13289.22 | -10862.70 | 1714.17 | 0.13 |
| 5 | -47154.67 | 18966.95 | -12576.87 | 7623.12 | 0.40 |
| 6 | -67354.66 | 35080.51 | -20199.99 | 3058.12 | 0.09 |
| 7 | -90612.77 | 63561.66 | -23258.11 | 147.77 | 0.00 |
| 8 | -114018.65 | 59652.37 | -23405.88 | 2935.32 | 0.05 |
| 9 | -140359.85 | 82054.53 | -26341.20 | 17579.89 | 0.21 |
| 10 | -149121.16 | 99840.02 | -8761.31 | NA | NA |

Supplementary Figure S1: Boxplots of male morphometric data of three subspecies of *Sporophila torqueola*, including (A) PC1, (B) PC2, (C) culmen length (mm), (D) length from gonys (mm), (E) bill depth at base (mm), (F) bill width at base (mm), (G) tarsus length (mm), (H) hallux length (mm), central rectrix length (mm), wing chord length (mm). Each color corresponds to a different subspecies, as shown in the key below. Sample sizes are displayed for each subspecies alongside a plate reproduced with permission from Lynx Edicions.


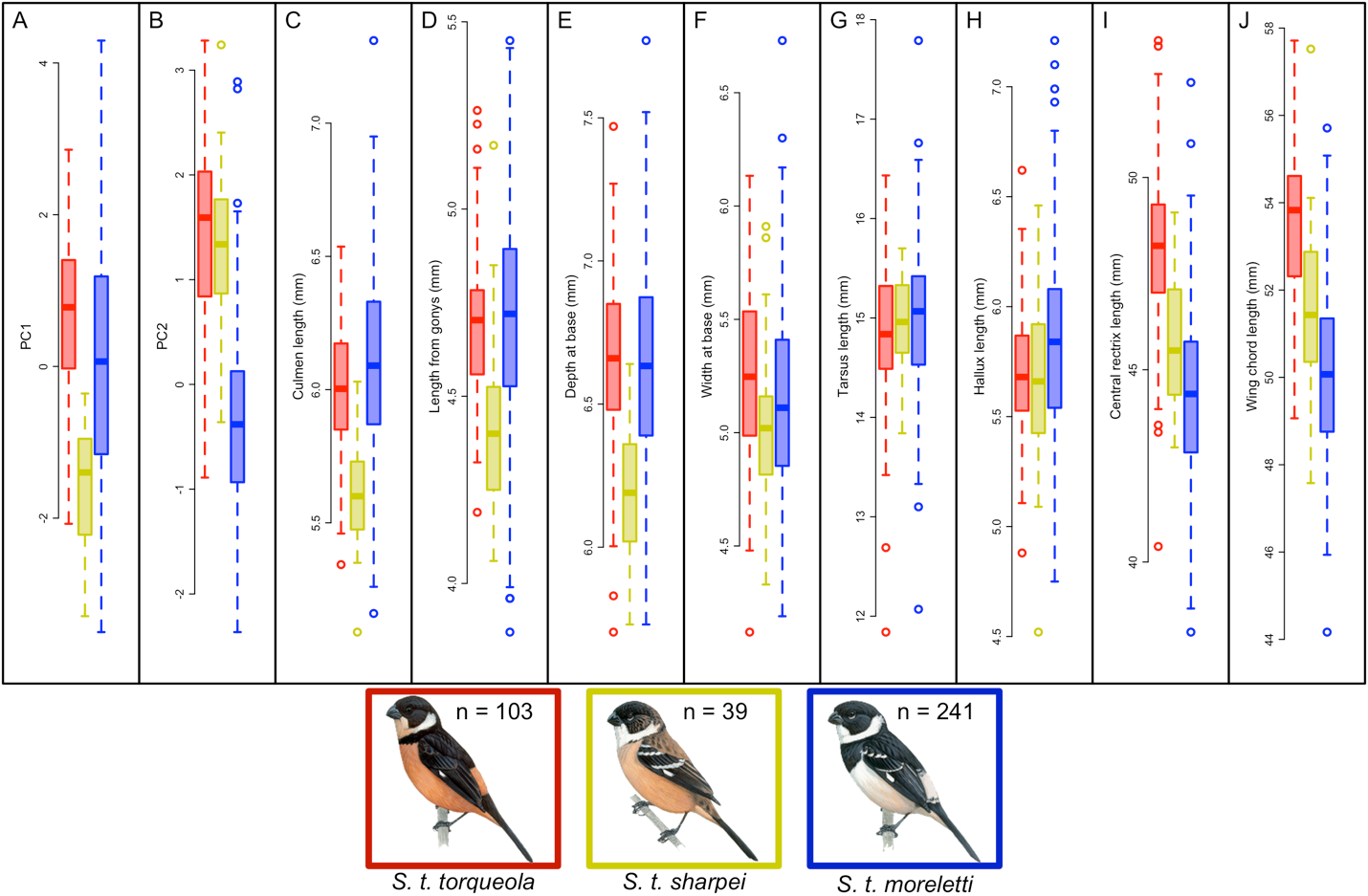
Supplementary Figure S2: Boxplots of female morphometric data of three subspecies of *Sporophila torqueola*, including (A) PC1, (B) PC2, (C) culmen length (mm), (D) length from gonys (mm), (E) bill depth at base (mm), (F) bill width at base (mm), (G) tarsus length (mm), (H) hallux length (mm), central rectrix length (mm), wing chord length (mm). Each color corresponds to a different subspecies, as shown in the key below. Sample sizes are displayed for each subspecies alongside a plate reproduced with permission from Lynx Edicions.


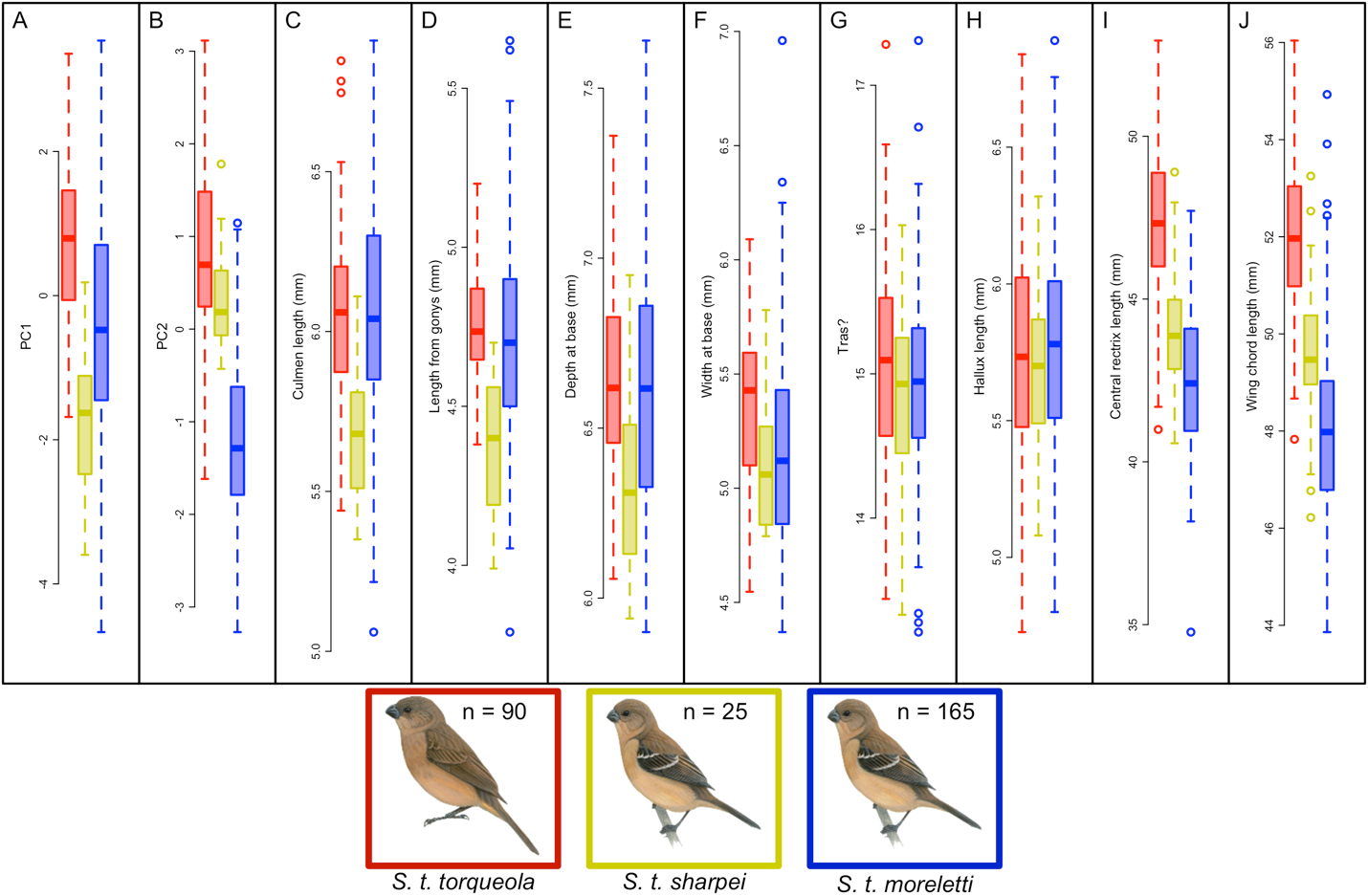


Supplementary Figure S3: Summary of UCE assembly statistics for 4376 recovered loci including (A) histogram showing lengths of UCE loci and (B) number of single nucleotide polymorphisms (SNPs) for UCE loci. 216 UCE loci were uninformative (i.e. contained zero SNPs).


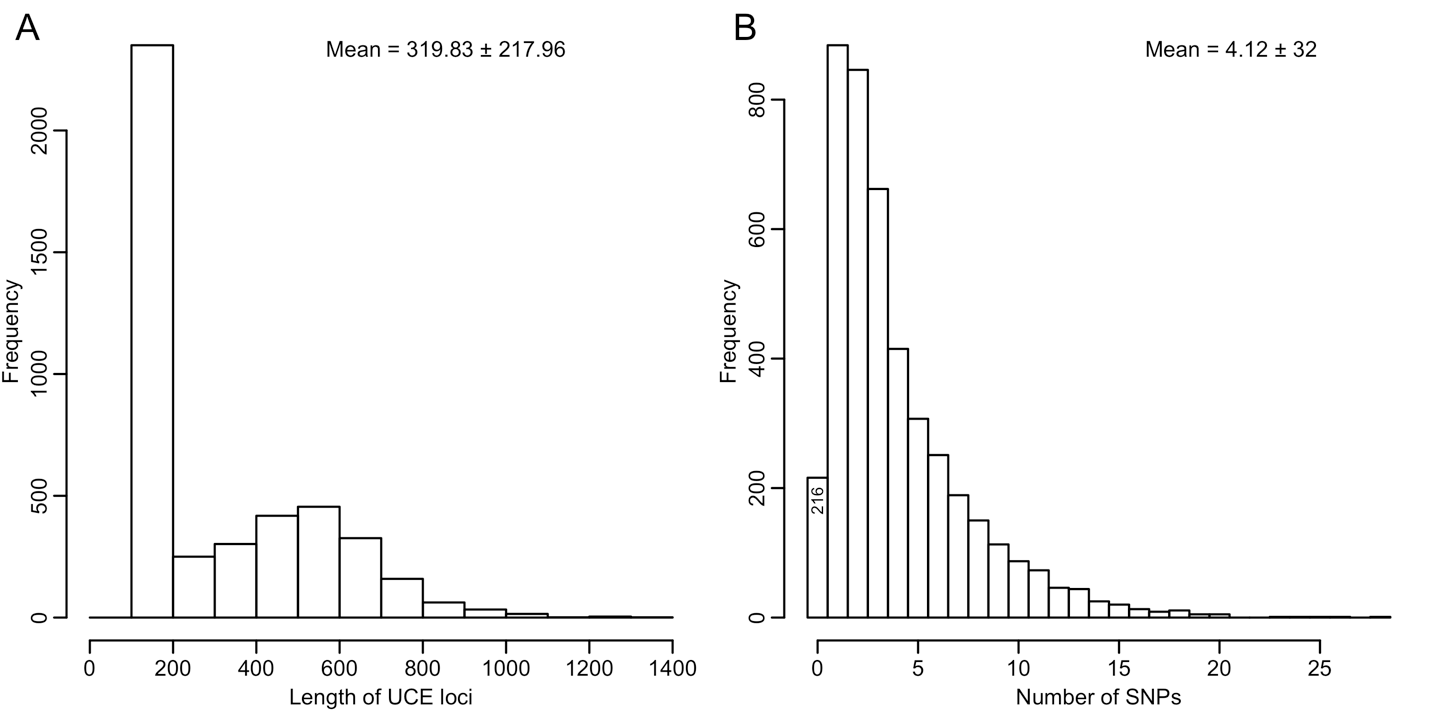


Supplementary Figure S3: Missing data from alignments of SNPs. One SNP was extracted per UCE locus for use in downstream analyses, resulting in 3154 possible SNPs. Here, we examine the amount of missing data for each locus (A) and each individual (B) in the resulting data set.


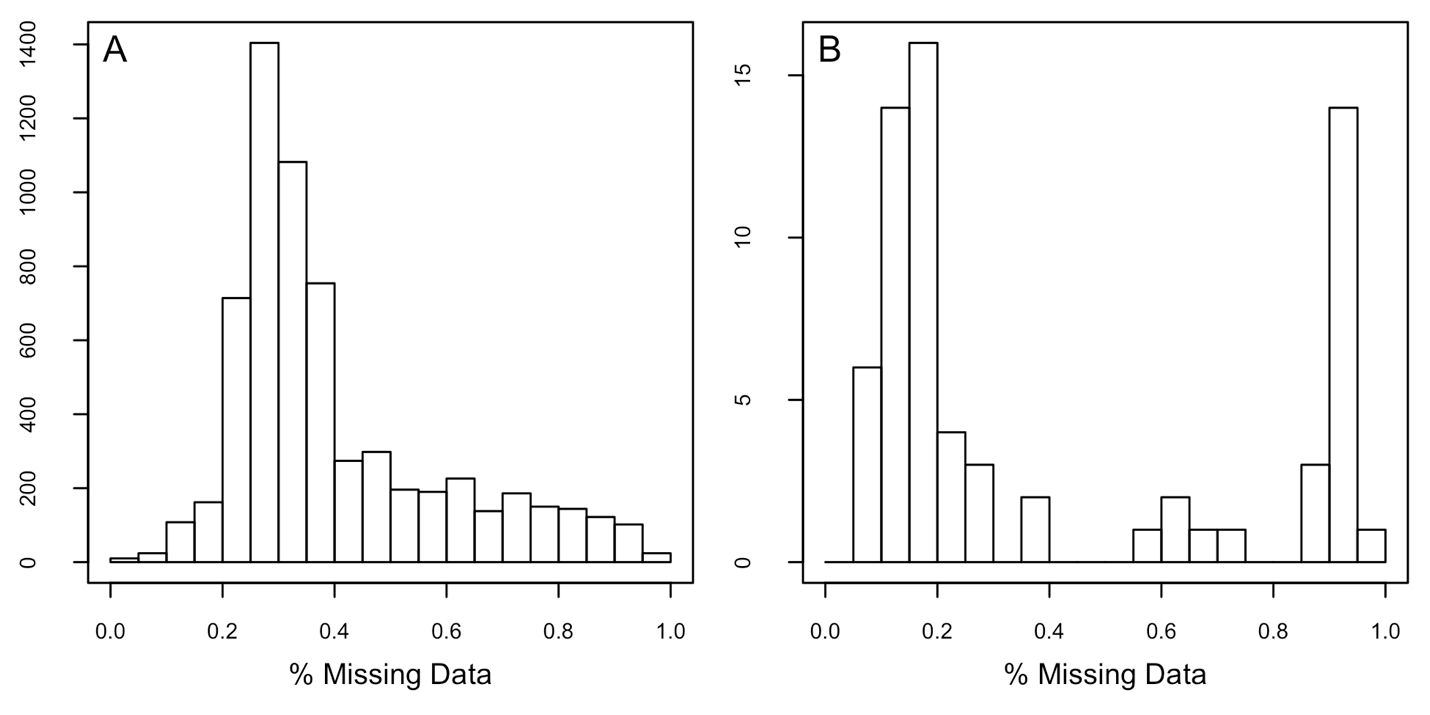


Supplementary Figure S4: Phylogenetic and population genetic analyses of full data set. (A) RAxML phylogeny built with 1000 loci with highest number of parsimonious sites. Built by searching for the best tree and performing rapid boostrapping in the same run (-f a setting). Nodes with white circles have bootstrap support above 70. Tip colors correspond to taxa in the lower right corner of the figure. (B) RAxML phylogeny of cyt *b* mtDNA sequences. Built by searching for the best tree and performing rapid boostrapping in the same run (-f a setting). Nodes with white circles have bootstrap support above 70. Tip colors correspond to taxa in the lower right corner of the figure. (C) PCA plot constructed by filtering data set to include individuals with less than 85% missing data (n = 54) and loci with less than 25% missing data (n = 1358). Dot colors correspond to taxa in the lower right corner of the figure. (D) STRUCTURE plot with optimal K value (3) determined by the Evanno method. Individuals are sorted according to taxa, with rectangular boundary colors corresponding to taxa in the lower right corner of the figure.


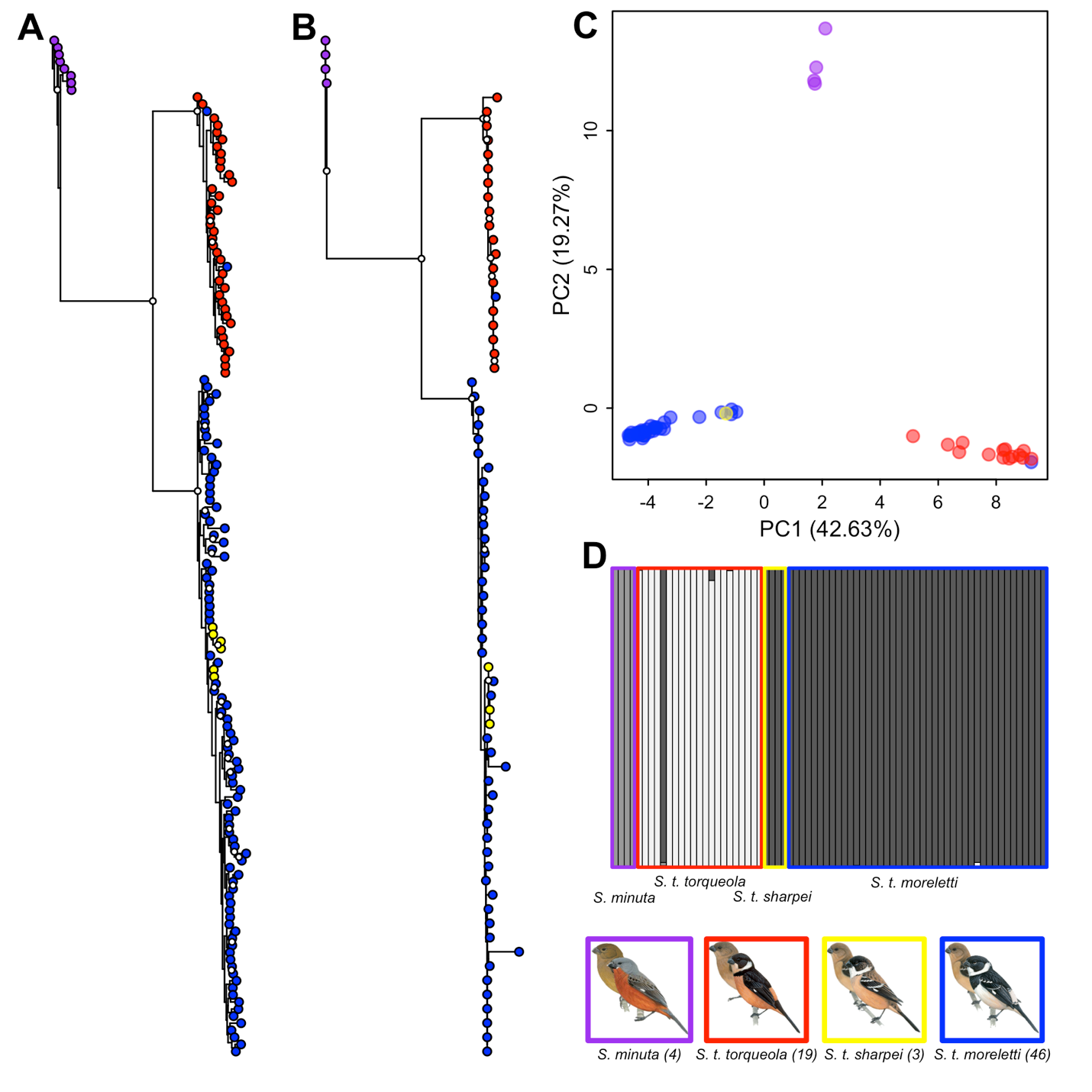
Supplementary Figure S5: Phylogeny of mitochondrial DNA of focal individuals and related taxa inferred with RAxML. Tips that have colored circles next to tip labels indicate individuals that were sampled in this study. The four purple circles represent females that were used as an outgroup in this study. Red circles correspond to *S. t. torqueola*, blue circles correspond to *S. t. morelleti*, and yellow circles correspond to *S. t. sharpei*. Tip labels include information about the country and state of collection. Node labels with white circles are nodes that received greater than 70 bootstrap support.


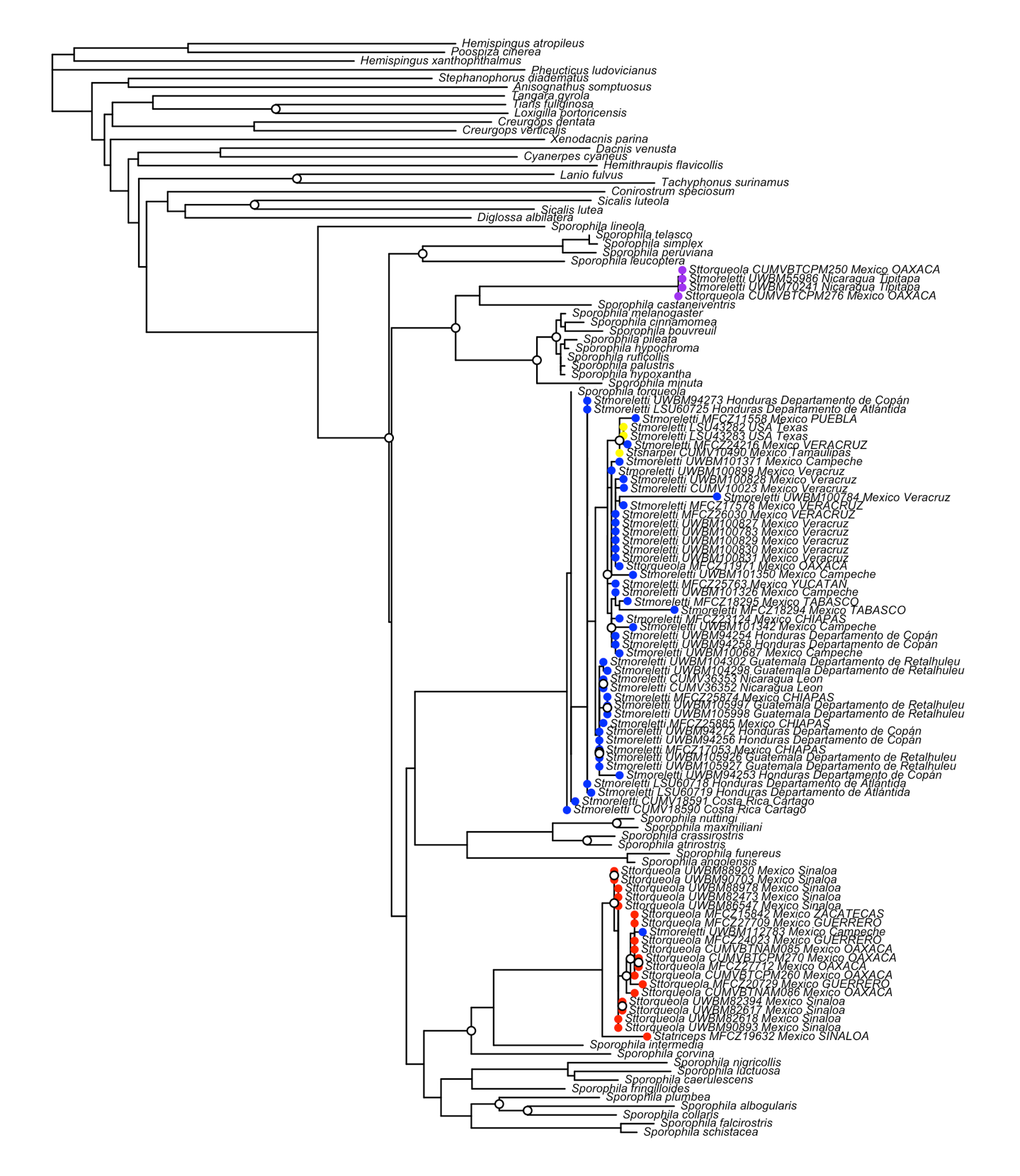


Supplementary Figure S6: Output from maximum likelihood search of ancestral states estimates using BioGeoBEARS. (A) MCC phylogeny with extant ranges for each tip in the phylogeny. Each node is numbered and corresponds to rows in panel B. (b) Probabilities for each possible ancestral range for each node. Only ancestral states with probability ≥ 0.05 for at least one node are shown (n = 28). Thus, all rows may not sum to equal exactly 1.0. (C) Areas used in BioGeoBEARS analysis and corresponding colors used in all panels.


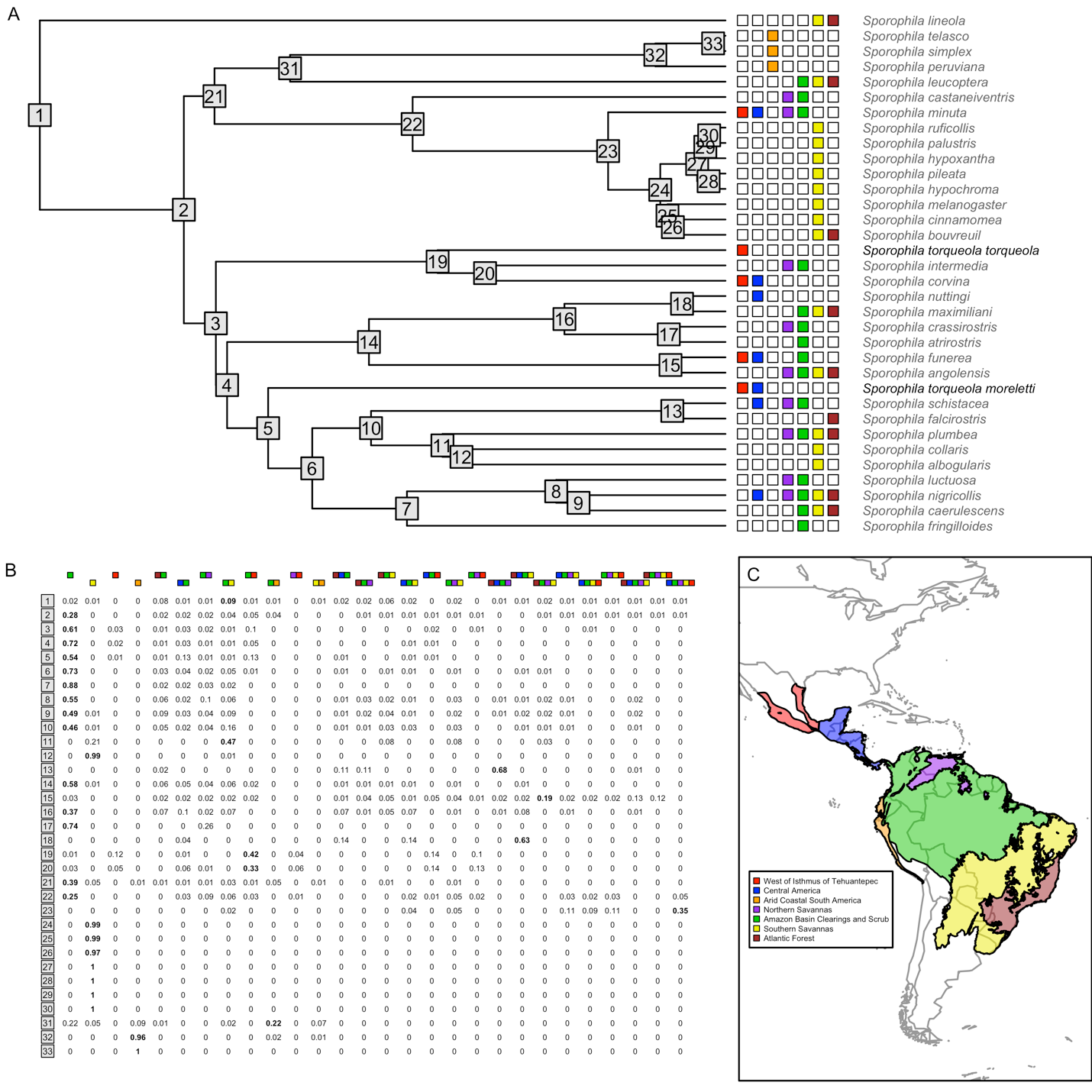

Supplement: Supplementary file 1 [file ECE3-8-1867-s001.docx]
